# Controlled efficacy trial confirming toltrazuril resistance in a field isolate of ovine *Eimeria* spp.

**DOI:** 10.1186/s13071-018-2976-4

**Published:** 2018-07-05

**Authors:** Ane Odden, Heidi L. Enemark, Antonio Ruiz, Lucy J. Robertson, Cecilie Ersdal, Silje K. Nes, Vibeke Tømmerberg, Snorre Stuen

**Affiliations:** 1 Faculty of Veterinary Medicine, Department of Production Animal Clinical Sciences, Norwegian University of Life Sciences, Kyrkjevegen 332/334, 4325 Sandnes, Norway; 2 Department of Animal Health and Food Safety, Norwegian Veterinary Institute, P.O. Box 750 Sentrum, 0106 Oslo, Norway; 3 Parasitology Unit, Department of Animal Pathology, Faculty of Veterinary Medicine, University of Las Palmas de Gran Canaria, 35416 Arucas, Las Palmas, Spain; 4 Faculty of Veterinary Medicine, Department of Food Safety and Infection Biology, Norwegian University of Life Sciences, P.O. Box 369 Sentrum, 0102 Oslo, Norway; 5 Animalia, Norwegian Meat and Poultry Research Centre, P.O. Box 396, Økern, 0513 Oslo, Norway

**Keywords:** Controlled efficacy test, Anticoccidial resistance, Toltrazuril, *Eimeria* spp., *Eimeria ovinoidalis*, Sheep

## Abstract

**Background:**

Coccidiosis due to *Eimeria* spp. infections in lambs causes increased mortality and substantial production losses, and anticoccidials are important for control of the infection. Anticoccidial resistance has been reported in poultry and swine, and we recently described reduced toltrazuril efficacy in ovine *Eimeria* spp. in some Norwegian sheep farms using a newly developed faecal oocyst count reduction test (FOCRT). The aim of the present study was to use a controlled efficacy trial to assess the efficacy of toltrazuril against a field isolate suspected of being resistant.

**Methods:**

Twenty lambs, 17–22 days old and raised protected against exposure to coccidia, were infected with a field isolate of 100,000 *Eimeria* spp. oocysts. This isolate was obtained from a farm with a previously calculated drug efficacy of 56% (95% confidence interval: -433.9 to 96.6%). At day 7 post-infection, 10 of the lambs were orally treated with 20 mg/kg toltrazuril (Baycox Sheep vet., Bayer Animal Health), while the other 10 lambs (controls) were given physiological saline. Clinical examinations were conducted, and weight gains recorded. Daily faecal samples were scored for diarrhoea on a scale from 1 to 5, and oocyst excretion was determined using a modified McMaster technique. Oocysts were morphologically identified to species level. At 17–24 days post-infection, the lambs were euthanized and necropsied.

**Results:**

The tested *Eimeria* isolate was resistant against toltrazuril, and resistance was seen in both pathogenic and non-pathogenic species. In addition, no significant differences in faecal score, growth, gross pathology or histological changes were identified between the two groups. The pathogenic *E. ovinoidalis* was the dominant species, and no significant difference in the individual prevalence of *E. ovinoidalis* post-treatment was found between treated (66.9%) and control lambs (61.9%). Other species identified included *E. crandallis*/*weybridgensis*, *E. parva*, *E. marsica*, *E. faurei*, *E. pallida*, *E. ahsata* and *E. bakuensis.*

**Conclusions:**

This study confirms toltrazuril resistance in ovine *Eimeria* spp.; in addition, the data support the use of FOCRT as an appropriate tool for field evaluation of anticoccidial efficacy. Due to limited anticoccidial treatment alternatives, these findings may have important implications for the sheep industry, particularly in northern Europe.

**Electronic supplementary material:**

The online version of this article (10.1186/s13071-018-2976-4) contains supplementary material, which is available to authorized users.

## Background

Anticoccidial resistance (ACR), which develops mainly as a result of intensive long-term use of anticoccidial drugs, occurs widely in poultry production and has also been identified in *Cystoisospora suis* in piglets [1–5]. In addition, a field method for the evaluation of reduced anticoccidial efficacy (ACE) in ovine *Eimeria* spp., the faecal oocyst count reduction test (FOCRT), has recently been developed and indicated that the efficacy of toltrazuril is reduced in some Norwegian sheep flocks [6].

Infections with *Eimeria* spp. may impact both animal welfare and productivity in the sheep industry, and controlling the infection is important to minimise mortality and morbidity, and to ensure that lamb growth is not compromised [7–9]. Suggested strategies to control ruminant coccidiosis include pasture management, adequate nutrition, and hygienic measures [10, 11]. However, these measures are often difficult to implement in practice, and the main control approach is often metaphylaxis with anticoccidials [12–15]. Metaphylactic administration of a single oral dose of toltrazuril in the prepatent period has been shown to be effective at reducing clinical signs and maintaining adequate lamb growth rates in different production systems [13, 15–19]. In contrast, treatment of clinical coccidiosis is considered inefficient due to the extensive intestinal damage already caused by the infection [20, 21]. Loss of sensitivity to toltrazuril, the only anticoccidial registered for use in sheep in the Nordic countries [22–24], should therefore be a matter for serious concern for lamb production.

The World Association for the Advancement of Veterinary Parasitology guidelines for evaluation of ACE in mammals [25], states that there is a need for verified methods for evaluation of ACE. Field methods for assessment of drug efficacy, such as the FOCRT [6] and the faecal egg count reduction test used to evaluate anthelmintic efficacy [26], give only an indication of reduced efficacy, and need verification through controlled efficacy trials (CET) [27, 28]. In addition, due to the variation in pathogenicity between ovine *Eimeria* spp., the differentiation of species should be considered separately [25].

The aim of the present study was to perform a CET in order to determine whether different species in a field isolate of ovine *Eimeria* spp. with suspected ACR, based on the FOCRT [6], actually demonstrated resistance to toltrazuril.

## Methods

### Study animals

A total of 20 lambs from 8 ewes of the Norwegian White Sheep breed (“Norsk kvit sau”) was included in the study, which was approved by the Norwegian Animal Research Authority (ID: 11657). The ewes were synchronised using Chronogest® CR and PMSG® (MSD Animal Health, Buckinghamshire, UK) and served by natural mating. Lambs were either snatched at birth (*n* = 16) or delivered by caesarean section (*n* = 4) over a period of 6 days, and thereafter reared artificially. Individual ear tags were used for identification. Directly after birth, all lambs were washed with Optima pH 4 soap (Optima Produkter AS, Norheimsund, Norway) and dried before being placed in boxes with expanded metal floors, in groups of four. Infrared heaters were used during the whole trial. An overview of the study groups, including lamb age, birth weight and gender can be found in Additional file 1: Table S1.

Lambs received ovine colostrum from ewes vaccinated against *Clostridium* spp. (Covexin-8, Zoetis) during the first 30 min of life, followed by colostrum from vaccinated cows (Covexin-8, Zoetis) during the next 24 h. To avoid cases of haemolytic anaemia, the cow-colostrum had previously been tested on naturally reared lambs. Lambs were then fed *ad libitum* with a commercial milk replacer (Denkamilk, Denkavit, Fiskå, Mølle, Stavanger), using an automatic feeding system (Holm & Laue, Godkalven, Figgjo, Norway). The lambs had *ad libitum* access to water, hay and commercial lamb-starter concentrate (FORMEL lam vår, Felleskjøpet, Norway). To ensure that transmission of *Eimeria* to the lambs *via* contaminated colostrum and hay could not occur, both were frozen at -75 °C for a minimum of 24 h, prior to provision to the lambs.

### Field isolate of *Eimeria*

The field isolate of *Eimeria* spp. was obtained from one of the flocks (ID 35) participating in the recent FOCRT study [6]. According to the FOCRT results, toltrazuril had reduced efficacy against *Eimeria* in two flocks. However, neither of these flocks were available for the CET, due to geographical and practical reasons. Thus, treatment with toltrazuril in the selected flock had been found to have an efficacy of 56.0%, but the results were classified as inconclusive, due to the wide 95% confidence interval (CI) of -433.9 and 96.6% [6].

To obtain sufficient *Eimeria* oocysts of this mixed field isolate (named “NMBU ID 35”) for the present study, faecal samples were obtained from 35 lambs in this flock 9 days after toltrazuril treatment (Baycox® Sheep vet., Bayer Animal Health, Oslo, Noray). Oocysts were isolated according to Jackson [29] with some modifications. Briefly, faeces were mixed 1:1 with water and filtered. The faecal mix filtrate was subsequently mixed 1:1 with saturated sugar-solution (density: 1.5 g/l) in a plastic container and left to float onto a glass slide. The slide was washed every second hour with deionized water for three consecutive days, and the washings collected. The washings were centrifuged at 2300× *g* for 20 min, the supernatant discarded and the sediment mixed 1:1 with deionized water in a glass flask with constant aeration. The oocysts in the flask were left to sporulate for 7 days at room temperature. Sporulated oocysts were stored for 18 days at 4 °C. Based on morphology [30], as seen by light microscopy at 400× magnification (see also Faecal samples section), and classification of 300 oocysts, the field isolate consisted of *E. parva* (32%), *E. crandallis*/*weybridgensis* (25%), *E. ovinoidalis* (24%), *E. faurei* (9%), *E. marsica* (8%), *E. pallida* (1%), *E. ahsata* (< 1%) and *E. bakuensis* (< 1 %).

### Infection and treatment of lambs

All lambs were infected (day 0) at 17–22 days of age, using an oesophageal tube. A dose of approximately 100,000 sporulated oocysts, diluted in water to a total volume of 5 ml, was given to each of the 20 lambs. Then, two randomly selected (coin toss) lambs from each group of four were orally treated (day 7) with 0.4 ml/kg toltrazuril (Baycox® Sheep vet. 50 mg/ml, Bayer Animal Health) and the remaining lambs (controls) were given 0.4 ml/kg of 0.9% NaCl (B. Braun Medical AS, Vestskogen, Norway).

### Body weight, general health and blood samples

Clinical examinations were performed daily throughout the trial. Rectal temperature was measured at days 0, 1, 2 and 7, and daily from day 14, and temperatures > 40.5 °C were considered as fever. The lambs were weighed once a week using a calibrated weight (Kruuse, Drøbak Norway) with 0.1 kg sensitivity, until 14 days post-infection, and thereafter three times a week.

Two lambs (controls) were treated orally with trimethoprim/sulphamethoxasole (Bactrim, Roche, Etterstad, Norway) during the first three days of life due to suspected *Escherichia coli*-infection, from which both recovered within 48 h. Six lambs, two controls and four treated with toltrazuril, developed lameness due to interdigital abscessation, and *Streptococcus aureus* was detected in two lambs. Four lambs recovered without treatment, and two of the lambs recovered after treatment with benzylpenicillinprocaine (Penovet vet., Boehringer Ingelheim Vetmedica, Copenhagen, Denmark) administered intramuscularly for three days.

On clinical examination, special attention was paid to clinical signs associated with *Eimeria* spp. infections, i.e. dehydration, pyrexia, weakness, anorexia and, in particular, the presence of diarrhoea.

Severe haemorrhagic diarrhoea and dehydration in one lamb at day 17, led to euthanasia of that whole group of four lambs. At day 18, another lamb showed signs of haemorrhagic diarrhoea, and all lambs in this group were also euthanized. The remaining three groups were euthanized on days 21, 23, and 24.

Blood samples were drawn from *v. jugularis* using vacuette tubes (plain and EDTA-treated; BD, Franklin Lakes, USA) at 48 ± 2 h after birth and at days 0, 7 and at euthanasia. Haematology was performed using the ADVIA 120 Haematology system (Bayer Diagnostics, Leverkusen, Germany). Dehydration was considered with a haematocrit (hct) of > 45.0% [31]. Whole blood tubes were centrifuged, and the serum removed and stored at -20 °C until further analysis. Biochemical analysis was performed by ABX Pentra 400 (Horiba, Les Ulis, France), and included analysis of iron, total protein, albumin, urea, creatinine, gamma-glutamyl transferase, glutamate dehydrogenase and beta hydroxybutyric acid.

### Faecal samples

Individual faecal samples from each of the lambs were obtained daily from day 10 of life until the end of the experiment. Visual scoring of faecal consistency was performed on a scale from one to five (1: normal, pelleted; 2: soft; 3: liquid; 4: watery; 5: watery with blood and/or intestinal tissue) [32]. A score ≥ 3 was considered as diarrhoea.

Samples were collected using an in-house “faecal spoon” [6] and the faecal samples were put in zip-lock bags, which were vacuum packed (Fresh’n’easy, OBH Nordica, Sundbyberg, Sweden), stored at 4 °C, and analysed within 37 days. The rate of oocyst excretion was determined using a modified McMaster technique with a theoretical sensitivity of 5 oocysts per gram (OPG) [6]. One hundred *Eimeria* oocysts from all samples ≥ 1000 OPG were examined by light microscopy at 400× magnification and identified to species level, using morphological criteria [30]. However, due to their morphological similarity, oocysts of *E. crandallis* and *E. weybridgensis* were not differentiated.

Oocyst counts were analysed by the FOCRT [6], which consists of a two-step procedure. First, timing of treatment and sampling was evaluated, followed by evaluation of treatment efficacy, by comparing post-treatment faecal samples from treated lambs with equivalent samples from untreated controls. Pre-treatment samples (sample 1) were obtained on day 7 (day of treatment), and post-treatment samples (sample 2) were obtained on days 14–18. The FOCRT was then run using the post-treatment oocyst counts for all five possible time intervals (7–11 days) between samples 1 and 2.

### Differential diagnoses

Faecal samples obtained at euthanasia were analysed for rotavirus, coronavirus, *Cryptosporidium* spp. and general bacteriology. Additional testing for *Cryptosporidium* spp. was performed in diarrhoeic lambs at the time of infection (day 0, *n* = 10). Faecal smears were analysed at the Norwegian Veterinary Institute in Oslo for *Cryptosporidium* by direct immunofluorescence analysis (Crypt-a-Glo™, Waterborne Inc., New Orleans, USA), whereas presence of rotavirus and coronavirus were tested by standard diagnostic methods. Samples for bacteriological analyses were obtained from mid-jejunum and the colon spiral, spread on sheep blood agar plates, and incubated under anaerobic and aerobic conditions for 24–48 h at 37 °C and 5% CO_2_. In cases of haemorrhagic diarrhoea, additional samples were grown on bromothymol-blue lactose cysteine agar (brolactin/CLED agar) for potential identification of *Salmonella* [33].

### Necropsy

Lambs were euthanized at days 17–24, by intravenous injection with pentobarbital (Euthasol vet., Virbac, Sollihøgda, Norway) at 140 mg/kg. Standard necropsy was performed immediately thereafter, with emphasis on the intestines.

Histological samples were taken from mid-jejunum, proximal and distal ileum, mid and base of caecum, colon spiral, and distal colon, in addition to heart, lung, liver and kidney. The samples were immersion-fixed in 4% formaldehyde, paraffin-embedded, and stained with haematoxylin and eosin (HE). Histological evaluation was performed by light microscopy and a blinded semi-quantitative evaluation (single evaluator) was done to assess intestinal pathology. Evaluation parameters included changes in: (i) villi, (ii) surface epithelium (atrophy/attenuation), (iii) degree of *Eimeria-*infection, (iv) hyperaemia, (v) oedema, (vi) infiltration of inflammatory cells and (vii) crypt abscesses, and were scored as follows: 0 = minimal; 1 = little; 2 = moderate; 3 = severe, including half-step grading. In addition, the presence of epithelial necrosis was graded as present (1) or absent (0). A total histology score was calculated for each tissue by summation of all parameters evaluated (i-vii).

### Statistical analysis

Data were managed in Excel 2013 (Microsoft Inc., Redmond, USA), and subsequently analysed in R [34] and Stata 14 (Stata Statistical Software: Release 14. StataCorp LP, College Station, TX, USA). Evaluation of efficacy was performed according to the FOCRT [6]. For calculations of significance based on means, a t-test was used. *P* < 0.05 was considered significant.

## Results

### Body weight, general health and blood analysis

**Fig. 1:**
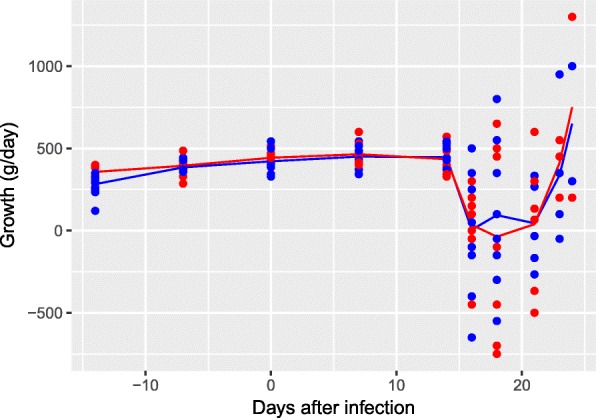
Mean and individual growth (g/day) of the 20 *Eimeria* spp*.* infected lambs. Red: toltrazuril treated, and blue: controls. *n* varies due to euthanasia: day ≤ 17, *n* = 20; days 18–20, *n* = 16; days 21–22, *n* = 12; day 23, *n* = 8; day 24, *n* = 4

Mean growth rates were above 300 g/day until days 14–16, whereupon mean growth rate decreased to around 0 g/day (Fig. 1). Growth rates increased again from day 21 onwards. The same pattern was observed in both treated and control lambs.

From day 15, both treated and control lambs had a mean faecal score of ≥ 3, indicating diarrhoea. The maximum mean faecal score was seen at day 17 (3.9 ± 0.2) and day 18 (4.4 ± 0.3) in the treated and control groups, respectively. Haemorrhagic diarrhoea was seen from day 14, in two treated and five control lambs, and tenesmus was observed in two control lambs (day 17).

An increase in rectal temperature was seen from day 14, with maximum temperatures measured at day 18 (40.4 ± 0.4 °C) and 16 (40.9 ± 0.4 °C) in the treated and control groups, respectively. The mean duration of fever (> 40.5 °C) was 2.3 ± 0.5 days and 1.9 ± 0.4 days for the treated and control groups, respectively. For these parameters, no significant difference between groups were seen at any time.

At euthanasia, the mean hct was 39.2 ± 1.7% and 41.4 ± 1.9% in the treated and control groups, respectively. However, dehydration (hct > 45.0%) was only seen in 3 lambs, of which one had been treated with toltrazuril. Mean total serum protein decreased in both groups from infection to euthanasia, but no significant differences between the groups were observed. Other biochemical parameters were within normal ranges (data not shown).

### Faecal analysis

**Fig. 2:**
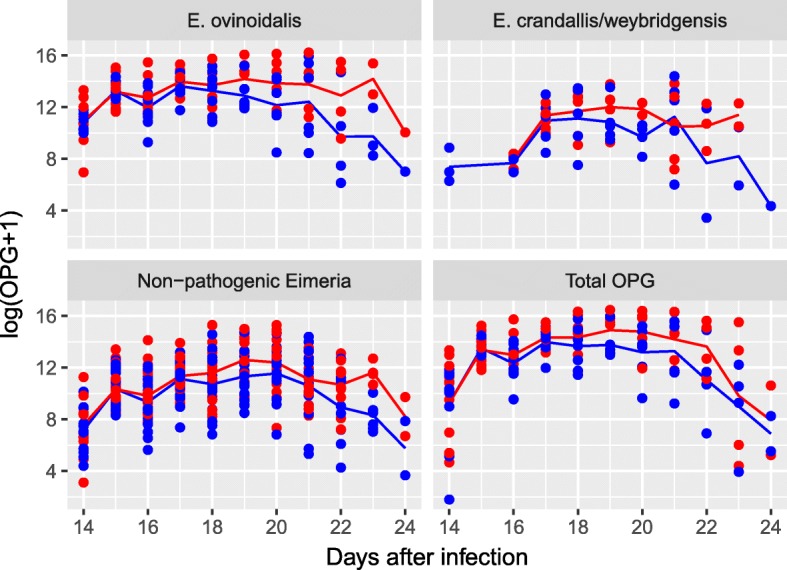
Mean and individual oocyst excretion [log(OPG+1)] in 20 *Eimeria* spp. infected lambs. *E. ovinoidalis*, *E. crandallis/weybridgensis*, non-pathogenic *Eimeria* spp. and the total OPG is shown. There was no significant difference in oocyst excretion between toltrazuril treated lambs (red) and controls (blue) at any time point. *n* varies: day ≤ 17, *n* = 20; days 18–20, *n* = 16; days 21–22, *n* = 12; day 23, *n* = 8; day 24, *n* = 4

Oocyst excretion was first recorded in one treated lamb at day 10 (10 OPG), followed by oocyst excretion in all lambs in both groups from day 14 onwards. Peak oocyst excretion was seen in the treated group at day 20 (mean OPG: 5,438,500), and in the control group at day 21 after infection (mean OPG: 3,630,850) (Fig. 2). Thereafter, oocyst excretion decreased. There was no significant difference in oocyst excretion and species distribution between the groups at any time. All species present in the field isolate were isolated from the faecal samples of all the 20 infected lambs. *E. ovinoidalis* was the most prevalent species in both treated and control lambs (Table 1).

**Table 1:** *Eimeria* spp. excreted by toltrazuril treated lambs (*n* = 10) and controls (*n* = 10). The excretion is presented as percentage per species of the total number of oocysts excreted

	Treated (%)	Control (%)
*E. ovinoidalis*	66.87	61.88
*E. crandallis/weybridgensis*	3.61	12.11
*E. faurei*	0.81	1.00
*E. pallida*	1.54	0.98
*E. parva*	23.51	19.83
*E. marsica*	3.60	4.12
*E. bakuensis*	0.02	0.00
*E. ahsata*	0.04	0.09

Efficacy, according to the FOCRT, was evaluated with confidence if the slope was ≥ 0.75, and with caution if slope was ≥ 0.5 and < 0.75 [6]. The slope ranged from 1.24 to 1.69 for the total oocyst excretion in the control lambs.

Slopes, maximum likelihood estimates, and 95% CIs for the geometric mean efficacy of all oocysts, *E. ovinoidalis*, *E. crandallis/weybridgensis*, and the non-pathogenic *Eimeria* spp. are presented in Table 2; reduced efficacy of toltrazuril is apparent against both pathogenic and non-pathogenic species. The slope was ≥ 0.75 for all time intervals and species, except for four of the five time intervals of *E. crandallis/weybridgensis*.

**Table 2:** Maximum likelihood estimates and 95% confidence intervals (CI) for the geometric mean efficacy

Sample 2 (day)	*Eimeria* spp.	Slope	Mean efficacy (%)	Lower 95% CI	Higher 95% CI	*n* ^a^		Interpretation
						Treated	Control	
14	All species	1.32	-0.3	-1116.8	92.5	10	10	Reduced efficacy
14	*E. c/w*	0.26	–	–	–	10	10	Invalid
14	*E. ovi*	1.33	-114.8	-431.4	16.8	10	10	Reduced efficacy
14	Non-pathogenic	1.22	-48.3	-252.3	44.0	10	10	Reduced efficacy
15	All species	1.69	13.5	-90.9	61.2	10	10	Reduced efficacy
15	*E. c/w*	0	–	–	–	10	10	Invalid
15	*E. ovi*	1.47	8.7	-102.8	63.2	10	10	Reduced efficacy
15	Non-pathogenic	1.31	33.0	-73.6	70.2	10	10	Reduced efficacy
16	All species	1.37	-93.4	-395.5	29.5	10	10	Reduced efficacy
16	*E. c/w*	0.26	–	–	–	10	10	Invalid
16	*E. ovi*	1.33	-114.8	-418.7	9.5	10	10	Reduced efficacy
16	Non-pathogenic	1.22	-48.3	-265.8	37.3	10	10	Reduced efficacy
17	All species	1.40	-41.9	-139.4	16.6	10	10	Reduced efficacy
17	*E. c/w*	0.73	-202,2	-834.6	-25.6	10	10	Caution: reduced efficacy
17	*E. ovi*	1.51	-41.2	-260.1	42.8	10	10	Reduced efficacy
17	Non-pathogenic	1.38	-37.0	-241.0	44.0	10	10	Reduced efficacy
18	All species	1.24	-97.2	-684.4	45.6	8	8	Reduced efficacy
18	*E. c/w*	0.77	-198.2	-769.8	-3.6	8	8	Reduced efficacy
18	*E. ovi*	1.47	-56.1	-316.6	47.6	8	8	Reduced efficacy
18	Non-pathogenic	1.35	-228.6	-815.1	-15.1	8	8	Reduced efficacy

*Notes*: The estimates were based on post-treatment oocyst counts for five time intervals between sample 1 (day 7 after infection) and sample 2, and was calculated according to the FOCRT [6]. A slope ≥ 0.5 and < 0.75 was evaluated with caution, whereas a slope < 0.5 was interpreted as invalid

^a^Four lambs were euthanized at day 17

*Abbreviations*: *E. ovi*, *E. ovinoidalis*; *E. c/w*, *E. crandallis/weybridgensis*; Non-pathogenic, all species except *E. ovinoidalis* and *E. crandallis/weybridgensis*

### Differential diagnoses

Samples analysed for *Cryptosporidium* spp., *Salmonella*, coronavirus and rotavirus were all negative. Bacteriological analyses showed a mixed flora, dominated by coliforms and *Enterococcus* spp.

### Necropsy

**Fig. 3:**
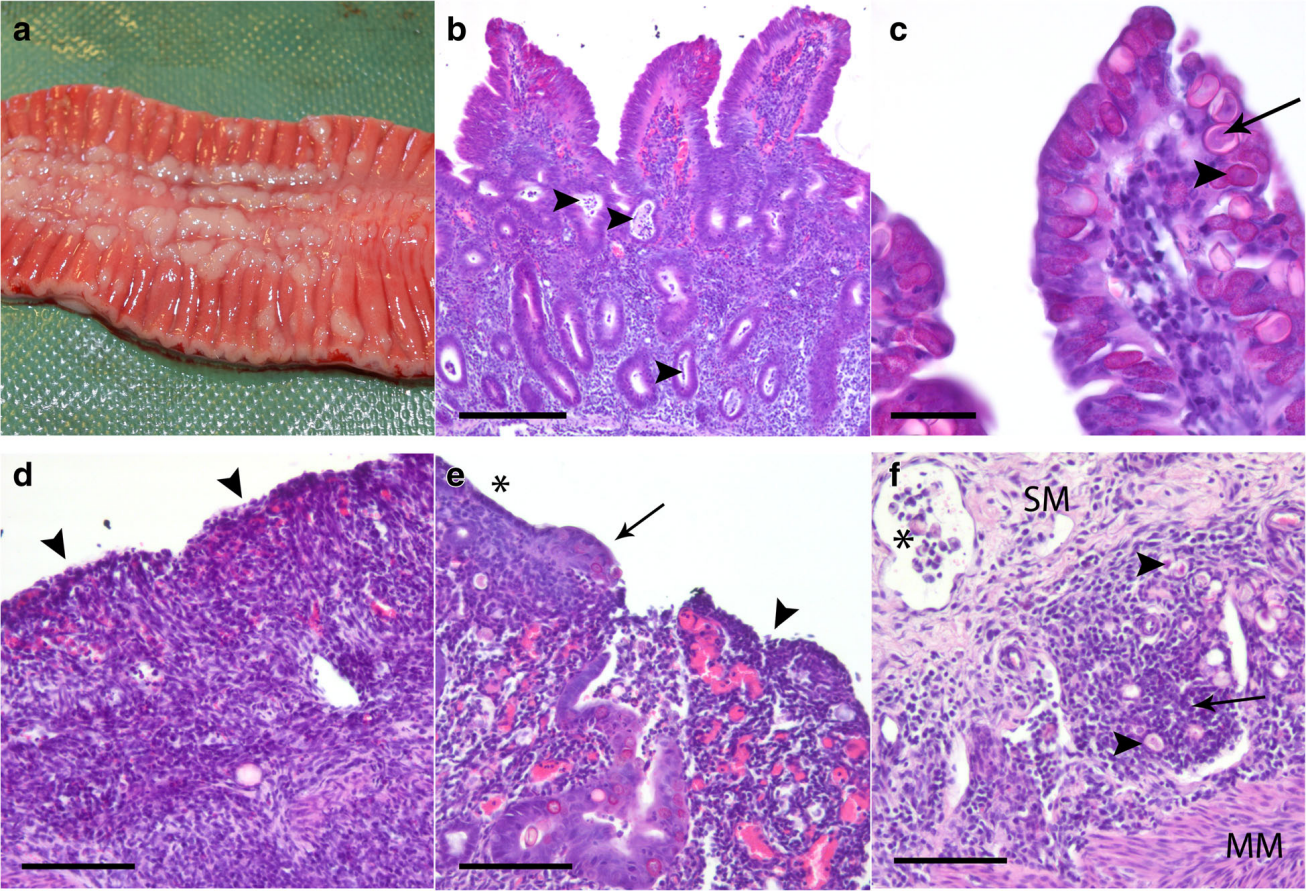
Examples of gross pathology and histological findings in lambs infected with *Eimeria* spp. **a**, **b**, **d-f** were treated lambs, while **c** was a control. **a** Section from ileum with multiple, coalescing beige nodules; also note the thickened and folded intestinal wall. **b** Proximal ileum: blunted villi with large amounts of *Eimeria* spp. in the epithelium. Arrowheads point at some of the numerous crypt abscesses. There is also infiltration of inflammatory cells in lamina propria and superficial haemorrhage and hyperaemia. **c** Heavy infection of surface epithelium of the proximal ileum with both gamonts (arrowhead) and zygotes (arrow) present. **d** Proximal ileum: large area of epithelial necrosis (arrowheads) with atrophy of villi and full destruction of normal architecture. There is marked infiltration of inflammatory cells, proliferation of fibrous tissue, hyperaemia and haemorrhage. **e** Basis of caecum: The surface epithelium is flattened (*), hyperplastic (arrow) and eroded (arrowhead). There is a colonic gland with hyperplastic epithelium and debris and next to this a destructed area with hyperaemia. **f** Basis of caecum: arrow points at a marked infiltration of inflammatory cells, mostly monocytes, with some *Eimeria*-zygotes (arrowhead) in submucosa (SM). A lymph vessel (*) with degenerated *Eimeria* (MM: muscularis mucosa). **b-f** Haematoxylin and eosin staining, scale-bars and magnification: **b**, 100 μm, 100×; **c**, 25 μm, 400×; and **d-f**, 50 μm, 200×

Gross pathological findings included diffused thickened and folded ileal mucosa (7 treated and 7 controls), and fibrinous ileal content in two lambs (one treated and one control). Nodular or plaque-like foci in the ileal mucosa were seen in 4 treated and 6 control lambs (Fig. 3a). The regional distal jejunal lymph nodes were moderately increased in size and oedematous in 5 treated and 6 control lambs. Finally, watery abomasal content was seen in > 50 % of the animals in both groups.

**Fig. 4:**
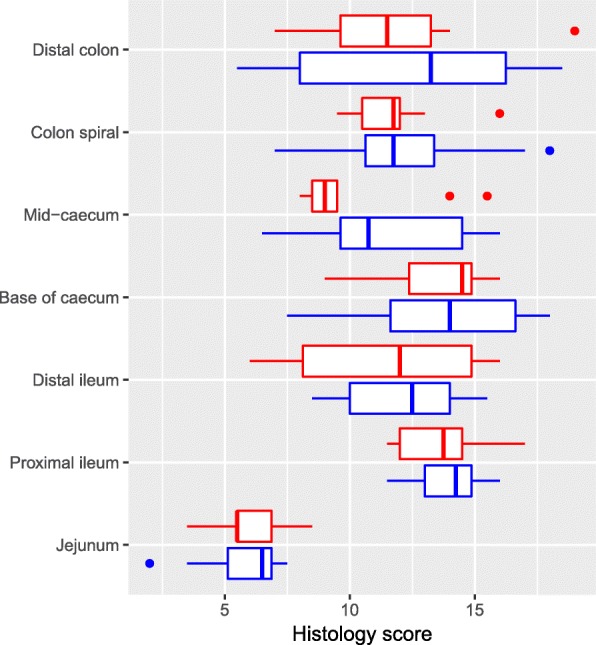
Box-and-whisker plots with outliers illustrating the histology score. The score was a summation of all histological parameters evaluated (see text) in the 20 *Eimeria* spp. infected lambs, red: toltrazuril treated, and blue: controls

Microscopy evaluation showed lesions, mainly in the ileum, caecum and colon, with minor lesions in the jejunum (Fig. 3b-f). However, there were no significant differences with respect to histological scores between the treated and control groups in any of the intestinal segments. The highest calculated histological score was found in the proximal ileum and at the base of caecum (Fig. 4). The mean score for each parameter can be found in Additional file 2: Table S2. Varying quantities of intracellular *Eimeria* stages were observed in all intestinal segments, except from jejunum, and they were mostly located in the villus epithelium, with fewer parasites in the crypt epithelium and lamina propria, and few in the submucosa and lymphatic vessels. In both treated and control lambs, changes in the intestinal surfaces varied from light atrophy of the jejunal epithelium and blunting of affected ileal villi (Fig. 3b), to areas of total flattening, attenuation of surface epithelium (Fig. 3e) and necrosis (Fig. 3d). Patches of epithelial necrosis were found in all lambs. Infiltration of inflammatory cells included mostly monocytes and eosinophils, but also neutrophils and macrophages, and was found in both the lamina propria and submucosa. Different degrees of oedema, hyperaemia, and haemorrhage were seen in all tissue sections examined, and in both treated and control lambs. Crypt abscesses (Fig. 3b) were found in varying degree in all lambs, and contained inflammatory cells, debris and different stages of *Eimeria* spp.

## Discussion

As far as we know, this is the first report of experimentally confirmed toltrazuril resistance in a field isolate of ovine *Eimeria* spp. The results also support the use of FOCRT as a tool to evaluate ACE in the field. Although ten of the 20 lambs experimentally infected with *Eimeria* were metaphylactically treated with the recommended dose of 20 mg/kg toltrazuril (Baycox® Sheep vet., Bayer Animal Health), this treatment did not result in a significant reduction in oocyst excretion in the treated animals, compared with the controls. In addition, no significant differences were noted in clinical presentation, gross pathology, and histopathological findings. The speciation data showed that both pathogenic and non-pathogenic species of *Eimeria* in this isolate were resistant to toltrazuril.

The lambs excreted high numbers of oocysts, as has previously been recorded in experimental infections with multiple *Eimeria* spp. [35]. Although oocyst excretion decreased from around day 20 after infection, the total duration of excretion could not be determined, as the lambs were euthanized. The excretion pattern noted here, with an exponential increase, a plateau phase, and a decline, has previously been noted in experimental infections [35–37]. However, due to continuous reinfection under natural field conditions, the duration of oocyst excretion may be longer [38, 39] than observed in the present study. This might also explain why the calculated slope seen for all species in this experimental study is higher than the slopes reported from the preceding field trial [6].

Multi-species resistance, as observed here, has also been noted in field isolates of avian *Eimeria* spp. [3, 40]. Of particular importance in this study is that *E. ovinoidalis* was the dominant species excreted from infected lambs. As this species is one of the most pathogenic *Eimeria* spp. in sheep [41, 42], resistance against the most commonly used anticoccidial drug indicates that severe clinical coccidiosis may be expected to occur in resistant flocks. Although *E. ovinoidalis* was the dominant species excreted, the most prevalent species in the original field-isolate inoculum was *E. parva*. This could reflect similarities between *E. ovinoidalis* and *E. ninakholyakimovae* in goats, the latter of which develops macroschizonts in endothelial cells, resulting in the release of thousands of merozoites [42, 43]. Thus, the extent of intracellular multiplication/replication, which is presumably also related to the extent of pathogenicity associated with this species, is higher for *E. ovinoidalis* than for the other *Eimeria* species.

For *E. crandallis*/*weybridgensis*, the FOCRT calculations showed invalid results from three of the five sampling time points, probably due to the tests being performed too early in the infection. Excretion of *E. crandallis/weybridgensis* increased predominantly from day 16 onwards, and euthanasia was performed at days 17–24. Thus, the longer prepatent periods for these species compared with *E. ovinoidalis* [44] probably explain these results. This is an important finding, as the number of invalid farms tested in the FOCRT [6] might have been fewer should sample 2 have been collected 10–11 days after sample 1. These findings also highlight the fact that although *Eimeria* spp. are often considered as a relatively uniform group, they are in fact separate species with potentially important differences in biology and pathogenic potential.

Two of the lambs were treated with trimethoprim/sulpha during their first days of life, preparations that have been shown to be effective in treating ovine coccidiosis [45, 46]. However, withdrawal periods for comparable drugs licenced in cattle are 10–15 days for meat [47], and these lambs were treated > 17 days prior to the experimental infection. In addition, these treated lambs were in the control group, and therefore this treatment should not have affected the results of the study.

Similar clinical signs as observed here might be caused by *Cryptosporidium* spp., coronavirus, rotavirus, and *Salmonella* spp., but none of these pathogens were detected. In addition, the findings of coliforms and *Enterococcus* spp. may be considered as normal intestinal flora of lambs [48]. The observed clinical signs were therefore almost certainly caused by *Eimeria* spp., particularly the two major pathogenic species, *E. ovinoidalis* and *E. crandallis* [35, 36]. Thickened ileal mucosa is often seen in lambs infected with *E. ovinoidalis* [49]. In addition, the histological changes, such as blunted villi and surface necrosis, as well as the presence of coccidia, hyperaemia, oedema, infiltration of inflammatory cells and crypt abscesses, are also in accordance with previous reports [42, 50, 51].

To improve our study, an additional group of uninfected lambs might have been advantageous as this would have enabled better comparisons between weight gain and histopathological changes. However, this was not feasible at the time of the study. Furthermore, due to the lack of defined cut-off values for ACE, it might have been advantageous to include an oocyst isolate from a non-suspected farm (i.e. a susceptible isolate) [25]. This would have enabled comparisons of different parameters, such as oocyst excretion, between treated and control lambs infected with susceptible or resistant *Eimeria* spp. However, due to lack of tools for selection of such susceptible ovine *Eimeria* isolates, we therefore chose to restrict our CET to treated and control lambs infected with isolate “NMBU ID 35” as a first step in the characterisation of anticoccidial resistance in ovine *Eimeria* spp.

Although the initial efficacy values have not been provided for toltrazuril by the manufacturer, several studies have investigated its effect on oocyst excretion. For example, its efficacy has been found to be 96.9–99.9% in the period from 7 to 98 days after first treatment, in a study in which the lambs were treated every 14 days [52]. Other studies have shown toltrazuril efficacies [either provided in the publication or calculated as 1-(mean OPG treated group)/(mean OPG control group) from data in the publication] ranging from 90.0 to 100.0% in the period from two to three weeks after treatment [13, 18, 19, 53–56]. These efficacies are far higher than that calculated in the present study, and therefore the comparative data provides a further clear indication of resistance in the “NMBU ID 35” isolate.

Toltrazuril has been marketed for anticoccidial treatment in sheep since the 1980s, and its use has increased during recent years, both in Norway [57] and in the UK (Dr Gillian Diesel, personal communication). Extensive use of a drug over time may result in decreased efficacy, possibly due to the haploid stages of *Eimeria*, which immediately select for resistance [1, 5]. Since toltrazuril is the only registered anticoccidial for sheep in several countries, development of resistance in ovine *Eimeria* species may result in there being few treatment options available for sheep farmers, especially in northern Europe [22–24]. Diclazuril is an anticoccidial that has been registered for treatment of sheep in several countries, but as it may share a common mode of action to that of toltrazuril [58], cross-resistance between these two triazine-derivates in ovine *Eimeria* spp. seems highly likely and should be investigated. Indeed, cross-resistance between diclazuril and toltrazuril was reported for an isolate of avian *Eimeria* spp. over 20 years ago [3].

Our results indicate that there is a clear need for tools for evaluating ACE, such that inefficient treatments and, thus, the potential for reduced animal welfare and productivity can be avoided. Such tools are available for poultry, using different metrics, such as oocyst index, body weight gain, relative weight gain, lesion scores and anticoccidial index [59]. However, such methods have not yet been established for use in ruminants [25], with the exception of the newly published FOCRT [6]. Although FOCRT may serve as a tool for field evaluation of ACE, there is a clear requirement for further testing of its use in different settings.

Confirmation of the spectre of resistance in ovine *Eimeria* species increases the urgency of identifying alternative treatments and optimising other control strategies. The anticoccidial effects of different plants and natural extracts, such as sainfoin (*Onobrychis viciifolia*), carob pods (*Ceratonia siliqua*), pomegranate (*Punica granatum*) peel extract, grape seed proanthocyanidin extracts, and different natural antioxidants, have been investigated *in vivo* and *in vitro* in different hosts [60–64]. However, none of these bioactive substances have, as yet, been brought to the market for the prevention of clinical coccidiosis. In addition, there are vaccines available for avian *Eimeria* spp. [65, 66], and successful immunisation of goat kids with attenuated *Eimeria* spp. oocysts has been performed [67].

Future studies are necessary in order to develop a commercial vaccine against ovine *Eimeria* spp. Therefore, current efforts should focus on identifying ACE, and maintaining the efficacy of toltrazuril in susceptible flocks. Management strategies that decrease the need for anticoccidials by reducing the infection pressure, possibly achieved by applying strict hygienic measures, and improved flock and pasture management should be actively encouraged by veterinarians and agricultural policy incentives [11]. Additionally, farmers should be informed about the importance of correct drenching techniques, including dosage estimation and drench gun calibration, as these have been shown to be inadequate in several farms [12].

## Conclusions

To our knowledge, this is the first report of ACR against toltrazuril in an ovine *Eimeria* field isolate, which included the highly pathogenic species, *E. ovinoidalis*. The results also support the use of FOCRT for field evaluation of ACE. However, the distribution and prevalence of ACR is unknown and further studies are warranted. In the future, difficulties in managing coccidiosis without chemotherapy, due to few available treatment options, may severely affect both animal welfare and the economy of the sheep industry.

## Additional files

Additional file 1: **Table S1.** Information about the 20 lambs infected with *Eimeria* spp. at day 0. (PDF 22 kb)

Additional file 2: **Table S2.** Histopathological findings from toltrazuril treated lambs and controls euthanized 17–24 days post-infection with 100,000 *Eimeria* oocysts. (PDF 118 kb)

## Abbreviations

ACE: anticoccidial efficacy
ACR: anticoccidial resistance
CET: controlled efficacy trial
FOCRT: faecal oocyst count reduction test
hct: haematocrit
OPG: oocysts per gram
