# Supplementary material for: Controlled efficacy trial confirming toltrazuril resistance in a field isolate of ovine Eimeria spp
Source: Parasit Vectors. 2018 Jul 5;11:394. doi: 10.1186/s13071-018-2976-4 (PMC6034276; doi:10.1186/s13071-018-2976-4)
Supplement: Supplementary file 1 — Table S1. Information about the 20 lambs infected with Eimeria spp. at day 0. (PDF 22 kb) [file 13071_2018_2976_MOESM1_ESM.pdf]

Table S1

Information about the 20 lambs infected with *Eimeria* spp. at day 0.

Group	Lamb	Sex	Birthweight (kg)	Age at infection	Weight at infection (kg)	Treatment with toltrazuril	Euthanasia (day)
A	1	Ram	4,3	22	11,5	No	23
	2	Ram	4,3	22	13,0	Yes	23
	3	Ewe	4,7	22	13,1	Yes	23
	4	Ewe	3,8	22	11,6	No	23
B	5	Ram	3,6	22	10,5	Yes	18
	6	Ewe	4,1	22	13,1	No	18
	7	Ewe	5,1	22	14,3	Yes	18
	8	Ewe	3,6	22	11,5	No	18
C	9	Ram	4,3	21	12,4	Yes	21
	10	Ewe	4,6	21	12,7	Yes	21
	11	Ram	4,1	19	11,1	No	21
	12	Ewe	3,0	19	8,9*	No	21
D	13	Ram	4,2	20	12,0	Yes	17
	14	Ewe	4,0	20	12,8	Yes	17
	15	Ram	6,0	18	13,9	No	17
	16	Ram	3,9	18	10,2	No	17
E	17	Ewe	5,3	18	12,9	Yes	24
	18	Ram	4,8	18	11,7	No	24
	19	Ram	7,1	17	14,2	No	24
	20	Ram	7,8	17	15,5	Yes	24

*Lamb 12 had to be bottle-fed and occasionally tube fed the first five days of life. Consequently, the growth was low the first week (120.0 g/day), but improved the following week (371.4 g/day).
