# Supplementary material for: Controlled efficacy trial confirming toltrazuril resistance in a field isolate of ovine Eimeria spp
Source: Parasit Vectors. 2018 Jul 5;11:394. doi: 10.1186/s13071-018-2976-4 (PMC6034276; doi:10.1186/s13071-018-2976-4)
Supplement: Supplementary file 2 — Table S2. Histopathological findings from toltrazuril treated lambs and controls euthanized 17–24 days post-infection with 100,000 Eimeria oocysts. (PDF 118 kb) [file 13071_2018_2976_MOESM2_ESM.pdf]

Table S2

Histopathological findings from toltrazuril treated lambs and controls euthanized 17-24 days post infection with 100,000 *Eimeria* oocysts.

	**Jejunum**		**Proximal ileum**		**Distal ileum**		**Base of caecum**		**Mid-caecum**		**Colon spiral**		**Distal colon**	
	Treated	Control	Treated	Control	Treated	Control	Treated	Control	Treated	Control	Treated	Control	Treated	Control
Epithelial reaction	0.7 ± 0.1	0.7 ± 0.1	2.4 ± 0.2	2.4 ± 0.1	1.6 ± 0.3	1.8 ± 0.3	2.2 ± 0.1	2.3 ± 0.2	1.8 0.2	2.1 ± 0.2	2.2 ± 0.2	2.3 ± 0.2	2.3 ± 0.3	2.2 ± 0.3
Epithelial necrosis	0	0	0.6 ± 0.2	0.7 ± 0.2	0.2 ± 0.1	0.5 ± 0.2	0.7 ± 0.2	0.6 ± 0.2	0.1 ± 0.1	0.2 ± 0.1	0.1 ± 0.1	0.2 ± 0.1	0.4 ± 0.2	0.5 ± 0.2
Degree of *Eimeria*-infection	0	0	1.6 ± 0.4	1.7 ± 0.4	1.9 ± 0.4	1.6 ± 0.3	1.9 ± 0.2	2.0 ± 0.3	1.2 ± 0.3	1.4 ± 0.2	1.4 ± 0.1	1.5 ± 0.2	1.3 ± 0.3	1.6 ± 0.4
Hyperaemia	1.7 ± 0.2	1.7 ± 0.2	2.4 ± 0.1	2.6 ± 0.1	1.9 ± 0.2	2.2 ± 0.2	2.3 ± 0.1	2.4 ± 0.1	2.1 ± 0.1	2.2 ± 0.2	2.3 ± 0.1	2.2 ± 0.2	2.3 ± 0.1	2.2 ± 0.2
Oedema	2.3 ± 0.1	2.1 ± 0.2	2.5 ± 0.2	2.5 ± 0.2	1.9 ± 0.2	2.2 ± 0.2	2.0 ± 0.1	2.0 ± 0.2	1.3 ± 0.1	1.9 ± 0.2	1.7 ± 0.2	1.6 ± 0.3	1.9 ± 0.3	1.8 ± 0.3
Inflammatory cells	1.4 ± 0.2	1.3 ± 0.2	2.3 ± 0.2	2.4 ± 0.2	2.0 ± 0.2	2.2 ± 0.2	2.5 ± 0.2	2.4 ± 0.1	2.2 ± 0.2	2.2 ± 0.2	2.5 ± 0.1	2.4 ± 0.1	1.9 ± 0.2	2.3 ± 0.1
Crypt abscess	0.2 ± 0.1	0.1 ± 0.1	2.0 ± 00.2	1.7 ± 0.3	1.9 ± 0.2	1.9 ± 0.2	2.1 ± 0.2	2.2 ± 0.2	1.4 ± 0.4	1.7 ± 0.3	1.8 ± 0.2	2.1 ± 0.2	1.7 ± 0.2	2.0 ± 0.3
Total score	5.9 ± 0.5	5.8 ± 0.6	13.6 ± 0.6	13.8 ± 0.5	11.4 ± 1.2	12.2± 0.8	13.5 ± 0.8	13.7 ± 1.1	10.1 ± 0.9	11.5 ± 1.0	11.8 ± 0.6	12.2 ± 1.1	11.6 ± 1.1	12.4 ± 1.1
Total score range	3.5 – 8.5	2.0 – 7.5	11.5 – 17.0	11.5 – 16.0	6.0 – 16.0	8.5 – 15.5	9.0 – 16.0	7.5 – 16.0	8.0 – 15.5	6.5 – 18.0	19.5 – 16.0	7.0 – 18.0	7.0 – 19.0	5.5 – 18.5

Haematoxylin and eosin stained tissue sections were blindly scored semi-quantitatively on a scale from 0-3, with half grading, except for villus necrosis (present = 1 or absent = 0), total score was calculated by summation
